# Conducting Polymer Based Nanobiosensors

**DOI:** 10.3390/polym8070249

**Published:** 2016-06-30

**Authors:** Chul Soon Park, Changsoo Lee, Oh Seok Kwon

**Affiliations:** 1 Hazards Monitoring Bionano Research Center, Korea Research Institute of Bioscience and Biotechnology (KRIBB), 125 Gwahak-ro, Yuseong-gu, Daejeon 34141, Korea; cspark@kribb.re.kr; 2 Nanobiotechnology and Bioinformatics, University of Science & Technology (UST), 125 Gwahak-ro, Yuseong-gu, Daejeon 34144, Korea

**Keywords:** conducting polymer, nanobiosensors, human sense mimicking, polypyrrole, polyaniline, poly(3,4-ethylenedioxythiophene)

## Abstract

In recent years, conducting polymer (CP) nanomaterials have been used in a variety of fields, such as in energy, environmental, and biomedical applications, owing to their outstanding chemical and physical properties compared to conventional metal materials. In particular, nanobiosensors based on CP nanomaterials exhibit excellent performance sensing target molecules. The performance of CP nanobiosensors varies based on their size, shape, conductivity, and morphology, among other characteristics. Therefore, in this review, we provide an overview of the techniques commonly used to fabricate novel CP nanomaterials and their biosensor applications, including aptasensors, field-effect transistor (FET) biosensors, human sense mimicking biosensors, and immunoassays. We also discuss prospects for state-of-the-art nanobiosensors using CP nanomaterials by focusing on strategies to overcome the current limitations.

## 1. Introduction

Nanoscale materials have been attracting interest from all fields of research, such as solar energy, fuel cells, hydrogen storage, and drug delivery, owing to their large surface areas. In particular, conducting polymer (CP) nanomaterials have remarkable physical and chemical characteristics and great potential from which to produce cost-efficient, large-scale, lightweight, and flexible devices [1]. CP nanomaterials also have inherent advantages, such as easy surface modification, biocompatibility, and large surface areas; thus, they are excellent candidates for use in electronic and optoelectronic sensing systems [2,3]. Nanobiosensors based on CP nanomaterials, including DNA chips, aptasensors, field-effect transistor (FET) biosensors, and immunosensors, have exhibited high-performance sensing properties toward target biological species [4,5]. Although numerous studies have been published on the effects of CP nanomaterials in biosensing systems, several technological challenges remain to be overcome. The current limitations of conventional CP-based biosensors include their high detection limit, slow response, and low selectivity. Further in-depth studies related to the fabrication of larger surface areas, the introduction of efficient interfacing strategies, and the integration of CP nanomaterials into biosensing systems must be performed to realize next-generation nanobiosensors based on CP nanomaterials.

Herein, we present recent research efforts that have reported significant sensing performance using nanobiosensors and discuss the prospects of scientific and technological challenges for preparing next-generation biosensors. We focus on three CP nanomaterials—polypyrrole (PPy), polyaniline (PANI), and poly(3,4-ethylenedioxythiophene) (PEDOT)—because these are common conducting polymers that can be easily prepared.

## 2. Polypyrrole (PPy) Nanomaterials

PPy, which is formed by polymerization of pyrrole, is one of the most promising conductive copolymers owing to its facile synthesis and high conductivity. In the past, PPy nanomaterials, such as nanoparticles (NPs), nanotubes (NTs), core-shell nanomaterials, and hollow nanospheres, have been used as potential components in electronic devices and for chemical sensors. However, recently, PPy nanomaterials have also been used for biosensors because of their facile functionalization and environmental stability. In this section, we introduce several nanobiosensors using PPy nanomaterials and discuss their prospects.

### 2.1. Bioelectronic Noses and Tongues

**Figure 1:**
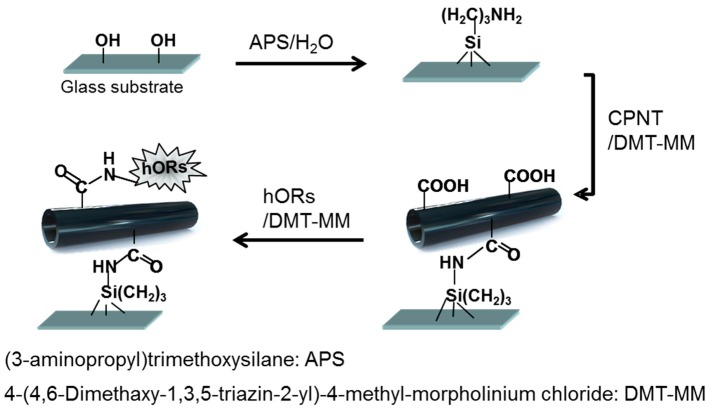
Fabrication process of the B-nose based on hOR-conjugated CPNTs. The hORs were immobilized on the surface of the CPNT by DMT-MM. CPNT: carboxylated PPy NTs; hORs: human olfactory receptors.

Mimicking of human senses is an important technique to realize novel smelling and tasting devices. Recently, Sang et al. [6] presented a carbon NT (CNT)-based bioelectronic nose (B-nose) that can discriminate at a single-carbon-atomic-resolution in an odorant mixture. Although the B-nose based on CNTs responded to amyl butyrate (ca. 100 fM), it had a limited sensitivity because the connection between the CNT and human olfactory receptors (hORs), which were expressed in *Escherichia coli* (*E. coli*) as recognition parts, was physical adsorption. To improve the performance of the B-nose, CP nanomaterials were investigated due to their facile functionalization. Jang et al. [7] fabricated PPy NTs using reverse microemulsion polymerization in an apolar solvent. The surface of the PPy NTs was easily modified with carboxylic groups by co-polymerization, and the carboxylated PPy NTs (CPNTs) were used for the B-nose. Yoon et al. [8] described the integration of hORs and CPNTs into a field-effect transistor (FET) platform that was controlled by a gating effect, resulting in a B-nose using CPNTs. The CPNT B-noses had covalent bonds between the hORs and transistors; thus, they were more stable in the liquid state than the conventional B-nose system and performed well in terms of odorant detection. The fabrication process of the B-nose based on CPNTs is shown in Figure 1.

With its aqueous-stable covalent bonding, the FET B-nose under a liquid ion gating system was characterized as p-type, which has holes as charge carriers. The liquid-ion gated FET B-nose exhibited highly sensitive real-time responses toward target molecules, and the minimum detectable level (MDL) was ca. 400 fM. Interestingly, the loading amount of hORs on the CPNTs was controlled by the covalent functionalization process, leading to an increasing MDL. This result indicates that the surface immobilization of hORs is an important factor in the performance of the B-nose. Whereas the previous study focused on detecting aqueous-state odorant mixtures, Lee et al. [9] demonstrated a human nose-like nanobioelectronic nose (NBE-nose) that responds to gaseous odorants. The NBE-nose had a sensing specificity response similar to that in the cellular signal transduction pathway. The NBE-nose also displayed an antagonistic behavior similar to that of a human nose.

CPNTs were also used for a mimicking nanobioelectronic tongue (NBE-tongue) by combining them with human taste receptors (hTRs: hTAS2R38s). Song et al. [10] prepared a FET NBE-tongue by the above method to demonstrate the mechanisms of human taste. The sensing performances with sensitivity and selectivity from NBE-tongue were highly similar to human nose. Moreover, the NBE-tongues had different responses to target bitter compounds depending on the taster and non-taster types of hTRs, and their MDL was ca. 1 fM for phenylthiocarbamide (PTC). The NBE-tongues showed different bitter-taste perception for compounds containing thiourea moieties in real vegetable samples and exhibited a concentration-dependent increase in drain-to-source current (*I*_SD_).

### 2.2. Aptasensors

**Figure 2:**
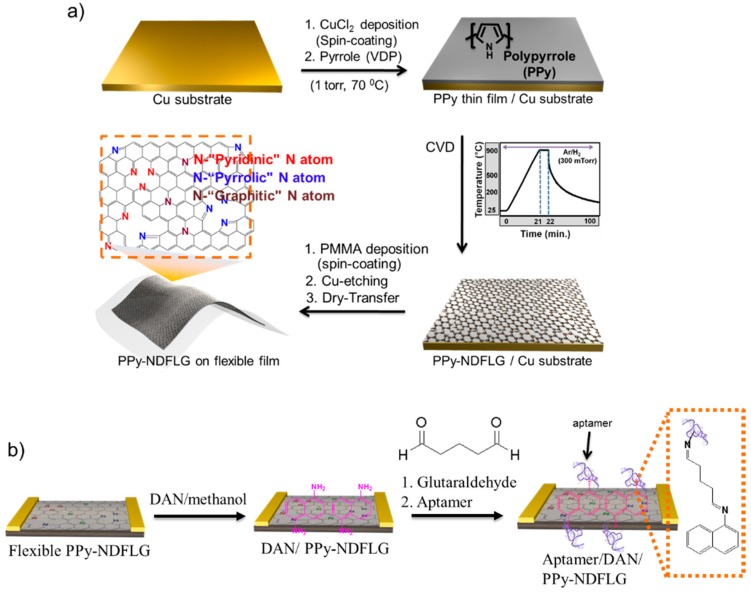
(**a**) Synthesis protocol of PPy-NDFLG on a flexible substrate; (**b**) Schematic illustration of the reaction steps for the fabrication of the aptasensor platform based on PPy-NDFLG conjugated with an anti-VEGF aptamer. Reprinted with permission from [11]. Copyright 2012, American Chemical Society. CVD: chemical vapor deposition; PPy-NDFLG: Polypyrrole-converted nitrogen-doped few-layer graphene; PMMA: poly(methyl methacrylate; DAN: 1,5-diaminonaphthalene; anti-VEGF: antivascular endothelial growth factor.

Aptamers, which consist of oligonucleotide or peptide molecules that bind to a specific target biomolecule, are promising bioprobes for biomolecule detection owing to their small size, chemical and environmental stability, and capacity for large-scale production. There are various nanomaterials for aptasensors, such as carbon-based nanomaterials, metal nanomaterials, ceramic nanomaterials, and CP nanomaterials. Kwon et al. [11] demonstrated nitrogen-doped few-layer graphene that was converted from 2D polypyrrole nanomaterials and integrated into FET aptasensors for antivascular endothelial growth factor (VEGF) detection. Polypyrrole-converted nitrogen-doped few-layer graphene (PPy-NDFLG) was fabricated by chemical vapor deposition combined with vapor deposition polymerization (Figure 2a). The PPy-NDFLGs were transferred onto a flexible substrate because of its high transparency, leading to flexible FET biosensors. The FET biosensor was fabricated by the adsorption of 1,5-diaminonaphthalene (DAN) on the surface of PPy-NDFLGs, and the aptamer was introduced onto the DAN/PPy-NDFLGs because of defect minimization (Figure 2b). Based on those immobilization processes of the aptamer, the field-induced high sensitivity from the FET PPy-NDFLG biosensors was observed for VEGF detection, and their MDL was ca. 100 fM. There is also considerable interest in the mechanical properties of the flexible FET PPy-NDFLG platform.

Interestingly, the aptamer can be incorporated into a PPy nanowire (PPy-NW) by a direct electrochemical deposition method. Huang et al. [12] demonstrated the one-step incorporation between an aptamer and PPy-NW-grown gold electrodes. The single PPy-NW with the aptamer was integrated into the fluidic substrate and had the responses toward IgE protein solutions in the range from 0.01 to 100 nM with excellent specificity and rapid stabilization times. The sensing mechanism of the PPy-NW aptasensor was a conductance change under a fixed voltage. The specific binding from the aptamer and IgE protein can increase the negative charge density on the PPy-NW because the IgE protein (with an isoelectric point of p*I* = 5.2–5.8) has a negative charge in pH 7.4 phosphate buffered saline (PBS) solution, leading to the conductance increase. The PPy-NW with an MUC1 aptamer also exhibited highly specific MUC1 biomarker detection under the fluidic system.

For the electrochemical aptasensor, Sun et al. [13] synthesized a PPy-Au nanocomposite (NC) using a one-step green synthesis method. The PPy-Au NCs provide a milder micro-environment and enlarged surface areas for myoglobin-binding aptamer (MBA) immobilization compared to bare PPy composite. The Au in PPy-Au NCs can physically bind to the –NH_2_ group of the MBA, and the electrochemical aptasensor responds to the myoglobin (Mb) in the biochemical assays over a wide detection range (0.0001 to 0.15 g·L^−1^). The MDL of the PPy Au NC aptasensor was 30.9 ng/mL.

### 2.3. Immunosensors

**Figure 3:**
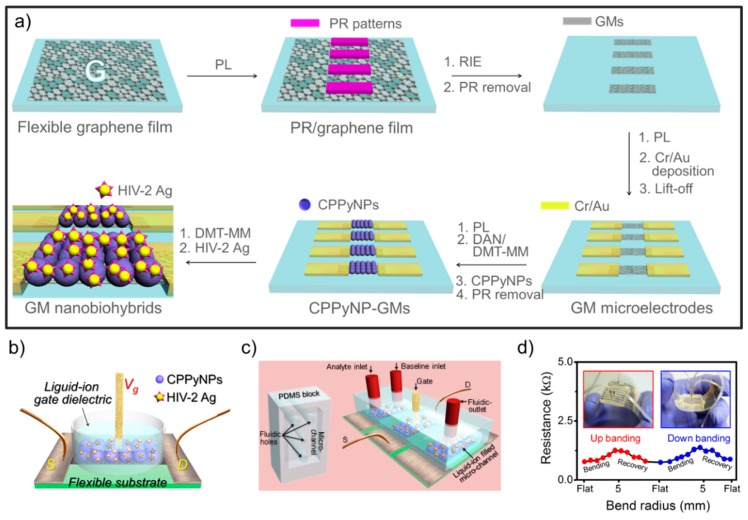
(**a**) Schematic illustration of the FET nanohybrid immunosensor based on CPPy NPs combined with graphene micropatterns on the flexible substrates; (**b**) Schematic illustration of the liquid-ion gated FET immunoassay; (**c**) Fluidic FET-type immunoassay, S: Source; D: Drain; (**d**) Variation in the resistance change of the flexible FET nanohybrids for different bending radius during and relaxing. Reprinted with permission from [17]. Copyright 2013, John Wiley & Sons Inc. (Hoboken, NJ, USA). PL: photolithography; PR: photoresistor; RIE: reactive ion etching; GMs: graphene micropatterns.

Immunoassays have been investigated as a major analytical tool for the diagnosis of diseases. In particular, antigen-antibody interaction is an attractive methodology for specific and selective sensing platforms. Recently, Tabrizi et al. [14] developed an electrochemical human immunoglobulin G (HIg G) immunosensor using overoxidized PPy decorated with gold NPs (OPPy-Au_nano_) owing to their excellent conductivity, stability, and biocompatibility. Specifically, the PPy was electrochemically deposited on the surface of the screen-printed electrode (SPE), and Au NPs were introduced by the same strategy. The OPPy-Au_nano_ film clearly showed high permeability to [Fe(CN)_6_]^3−/4−^. The Anti-IgG was immobilized on the surface of OPPy-Au_nano_/SPE by physical adsorption. The immunosensor displayed excellent ability to detect HIg G in the concentration range of 0.5 to 12.50 ng·mL^−1^ and had a MDL of 0.2 ng·mL^−1^. For another study using an electrochemical process for a PPy nanocomposite, Mishra et al. [15] reported an electrochemically synthesized ZnS nanocrystal-modified PPy NC film to detect the C-reactive protein (αCRP). The film showed high biocompatibility with efficient binding to the protein antibody (αCRP-Ab) and exhibited a linear impedance response to the αCRP-Ag concentration in the range from 10 ng·mL^−1^ to 10 µg·mL^−1^. Wang et al. [16] first fabricated multifunctionalized reduced graphene oxide (rGO)-doped PPy/pyrrolepropylic acid (PPa) NCs and applied them as an immunosensor. The NCs were prepared by the electrochemical polymerization of Py and Pa in the presence of rGO under a constant current technique. Each part of the NC possesses different properties for a high-performance immunosensor: (i) rGO is used for high conductivity; (ii) Pa provides covalent linkers; and (iii) PPy allows for the use of the film’s electroactivity from its inherent electrochemical doping/dedoping property for impedance measurements. The sensing platform using rGO/PPy/PPa NCs had been confirmed by cyclo voltammetry (CV) and electrochemical impedance spectroscopy (EIS) measurements and showed improved selectivity and reproducibility to detect aflatoxin B1 (AFB1) in the range of 10 fg·mL^−1^ to 10 pg·mL^−1^ with high specificity. This sensing platform represents a novel method to sensitively detect small molecules using an immunoassay technique. Although rGO incorporates high conductivity into the sensing platform, graphene grown on the Cu substrate by chemical vapor deposition (CVD) is also an attractive nanomaterial for CP nanohybrids. Recently, Kwon et al. [17] first demonstrated large-scale, flexible and fluidic FET-type HIV immunoassay devices using carboxylated PPy (CPPy) NPs/graphene micropattern nanohybrids. Specifically, the CVD-grown graphene was transferred onto the flexible polymer film and micropatterned by a photolithography process (microelectromechanical system: MEMS) with highly uniform sizes and shapes. To enlarge the surface areas, the CPPy NPs were immobilized on the surface of the graphene micropatterns by covalent bonding, and the HIV antigen was attached to the surface of the CPPy NPs (Figure 3a). The nanohybrids were integrated into the liquid-ion gated FET system, which is suitable for biosensors (Figure 3b). Moreover, to develop an immunoassay with portable, fluidic, and implantable characteristics, the poly(dimethylsiloxane) (PDMS) microchannels were designed by the MEMS process and integrated into the FET nanohybrid geometry (Figure 3c). The FET nanohybrids connected with PDMS microchannels exhibited HIV antibody detection (ca. 1 pM) with high selectivity and sensitivity. Importantly, the FET nanohybrids integrated with fluidic channels demonstrated real-time responses toward the injected HIV-2 gp36 antibody. The variation of the flexible nanohybrid film was also characterized by a repeated bending/unbending recycle process and displayed mechanical bendability and durability (Figure 3d).

### 2.4. H_*2*_O_*2*_ Biosensors

Hydrogen peroxide (H_2_O_2_), a reactive oxygen species, is related to several diseases, such as atherosclerosis, cancer, and Alzheimer’s disease, but is also essential for cell growth, migration, and the immune system. Thus, the development of H_2_O_2_ detection methods is highly challenging in the extensive field of healthcare and biological monitoring. Mahmoudian et al. [18] reported a facile method for synthesizing PPy-coated silver nanostrip bundles (Ag NSBs-PPy) via the direct reduction of Ag cations in the presence of the pyrrole monomer in an aqueous solution of AgNO_3_ and NaOH. This material was then used for H_2_O_2_ detection. The nanomaterials were integrated into an electrochemical system and exhibited remarkable catalytic performance for H_2_O_2_. In particular, the reaction with H_2_O_2_ at the glassy carbon electrodes increased owing to the enlarged surface areas of Ag NSBs-PPy. The sensor consisting of Ag NSBs-PPy has a rapid amperometric response time of less than 5 s, and its MDL was estimated to be 0.68 μ·mol·L^−1^. Nia et al. [19] also demonstrated H_2_O_2_ detection using PPy-coated copper NPs. Interestingly, they proposed electropolymerization and copper electrodeposition in a one-pot aqueous solution for preparing PPy micro trucks with CuNPs (PPyMTs-CuNPs). The structures, morphology, as well as the catalytic and electrochemical properties of the nanomaterials were characterized by various analyses, including field emission scanning electron microscopy (FE-SEM) and X-ray diffraction (XRD), and the PPyMTs-CuNPs showed increasing electrocatalytic activity toward the reduction of H_2_O_2_ (the MDL was 0.9 μm). Several studies used graphene for H_2_O_2_ detection. Park et al. [20] proposed a rapid-response and high-sensitivity H_2_O_2_ biosensor based on a PPy-embedded rGO transducer, which was introduced for a FET system. The rGO/PPy NTs showed p-type characteristics in a liquid-ion gated FET geometry. Their hole-transport behavior and conductivity are superior to those of rGO sheets or PPy NTs because of the formation of PPy NT bridges between the rGO sheets. The FET H_2_O_2_ biosensor had specific selectivity and rapid sensitivity toward H_2_O_2_ in a mixture consisting of compounds found in biological fluids. Recently, Tao et al. [21] discovered a novel function of PPy NPs, which is an intrinsic peroxidase-like activity. PPy NPs have been commonly used for drug delivery, tissue engineering, and photothermal therapy. However, surprisingly, they also catalyze the reaction of the peroxidase substrate 3,3′,5,5′-tetramethylbenzidine in the presence of H_2_O_2_ to produce a blue color. These results illustrate that the PPy nanomaterials enable the utilization of this intrinsic peroxidase activity in biotechnology.

### 2.5. Others

In various applications using PPy nanomaterials, one of the main challenges is the formation of an ultra-thin film because the CP thin layers provide facile surface modification and stable environments. Ghadimi et al. [22] proposed ultra-thin PPy nanosheets (UltraPPy) as modified electrodes, which were decorated with Pt NPs (Pt/UltraPPy-GCE) and integrated into an electrochemical system to detect dopamine (DA). The crosslinks of UltraPPy nanocomposites improve the stability of the Pt NPs, leading to excellent electrochemical activity toward the target molecules. Compared to bare GCE, the Pt/UltraPPy-GCE showed excellent sensing properties for DA detection due to the enlarged surface areas of the UltraPPy and Pt NPs, and its MDL was 0.67 nM. Moreover, the electrodes displayed good operational and storage stabilities for DA detection. Another challenge is associated with biosensor printing methods using PPy nanomaterials because printed biosensing platforms have low costs and production scalability. Weng et al. [23] presented a fabrication process for wholly printed PPy NPs on screen-printed carbon electrodes. This system consists of a flexible substrate used for a printable electrochemical biosensor chip by introducing two enzymes, horseradish peroxidase (HRP) or glucose oxidase (GoD), for H_2_O_2_ or glucose specificity. The sensing performances of wholly printed PPy NP biosensors were confirmed by cyclic voltammetry (CV) and chrono-amperometry. The MDLs of the biosensor were 10 μM–10 mM for H_2_O_2_ and 1–5 mM for glucose.

## 3. Polyaniline (PANI) Nanomaterials

PANI is one of the most promising conducting polymers, with enhanced conductivity, good environmental stability, and diverse color changes corresponding to different redox states. PANI nanomaterials can be applied in many practical fields as chemical sensors, supercapacitors, corrosion protection materials, batteries and energy storage devices, and antistatic coatings. Therefore, numerous studies have focused on the synthesis and application of PANI nanomaterials [24]. Unfortunately, the use of PANI nanomaterials in biological applications is limited by their low processability, lack of flexibility, and non-biodegradability. In addition, these materials have been noted to cause chronic inflammation once implanted [25,26]. PANI has been investigated for use in biosensors, neural probes, controlled drug delivery, and tissue engineering applications [27,28].

### 3.1. Glucose Biosensors

**Figure 4:**
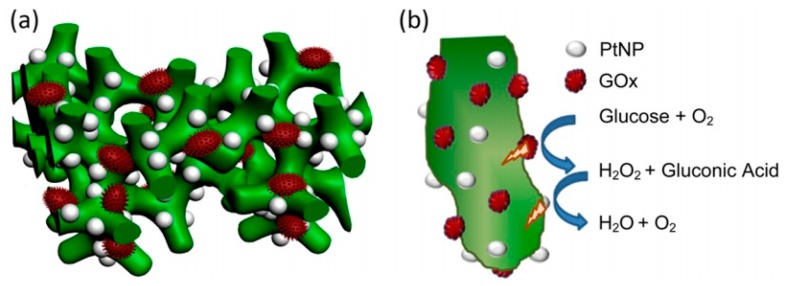
(**a**) Schematic representation of the 3D heterostructure of the PtNP/PANI hydrogel, in which the PANI hydrogel acts as a matrix for the immobilization of the GOx enzyme and homogeneous loading of PtNPs; (**b**) A 2D scheme showing the reaction mechanism of the glucose sensor based on the PtNP/PANI hydrogel heterostructure. Reprinted with permission from [41]. Copyright 2013, American Chemical Society. GOx: glucose oxidase.

Numerous glucose sensors have been proposed and developed over the past 30 years owing to the importance of glucose detection for patients suffering from diabetes. However, there are still several challenges associated with the development of a miniaturized device that can rapidly and reliably monitor glucose in vivo, independent of blood oxygen concentration. Traditionally, the electrochemical [29,30] or chemical synthesis [31,32,33] methods are used to synthesize conducting polymer-based layers on the electrode surface. In the process of electrochemical synthesis, conducting polymers can be easily doped by various biomaterials, such as enzymes and proteins. Electrochemical methods are most applicable for the preparation of films, but chemical [31] and enzymatic [34] methods are more useful for the preparation of conducting polymer-based NPs. As an alternative method, therefore, the enzymatic synthesis has been applied to the preparation of conducting polymers-based composite materials, including PANI [35,36,37,38,39]. Mazeiko et al. [40] developed two new nanocomposite structures: (i) glucose oxidase (GOx) entrapped within a PANI layer (GOx/PANI) and (ii) GOx and gold NPs (GNPs) entrapped within a PANI layer (GOx/GNPs/PANI). To compare certain parameters of the two different nanocomposite materials, enzymatic polymerization of PANI was performed in two types of polymerization solutions depending on presence or absence of GNPs. The first aniline polymerization solution was composed of four main components, containing aniline, glucose oxidase, glucose, and oxygen, dissolved in a buffer. In such a solution, the formation of H_2_O_2_, a strong oxidizing agent, was catalyzed by the GOx, which initiated the formation of PANI and encapsulation, or at least partial coverage, of GOx within the formed PANI layer. GNPs were contained in the next type of polymerization solution. The formation of PANI occurs more rapidly in the presence of GNPs than in the absence of GNPs. Zhai et al. [41] demonstrated highly sensitive glucose enzyme biosensor based on hydrogel heterostructure composed of Pt nanoparticles (PtNPs) and PANI. As shown in Figure 4, highly dense PtNPs were homogeneously immobilized on the 3D porous nanostructure of PANI hydrogel. The high density immobilization of the enzyme and the penetration of water-soluble molecules were assisted by the highly porous structure of the PANI hydrogel, which helped efficiently catalyze the oxidation of glucose. Furthermore, the PtNPs catalyzed the decomposition of H_2_O_2_ that was generated in the process of enzymatic reaction. The transferred charges from the electrochemical process were efficiently reduced by the highly conducting PtNP/PANI hydrogel heterostructures. The glucose enzyme sensor exhibited unprecedented performance with ultrahigh sensitivity (96.1 μA·mM^−^^1^·cm^−^^2^), fast response (3 s, a linear range of 0.01 to 8 mM), and a very low detection limit (0.7 μM).

PtNP/PANI hydrogel heterostructures were deposited on the carbon rod electrodes and investigated amperometrically. At all occasions, the GNPs promoted electron transfer (ET) and demonstrated a positive effect on the amperometric signals of all evaluated types of electrodes. The sensitivity, apparent Michaelis constants and various characteristics of prepared biosensors were also determined and investigated. The results demonstrate that GOx/PANI and GOx/GNPs/PANI nanocomposites maintain the catalytic activity of glucose oxidase and appear to be appropriate to the design of amperometric biosensors, predicting possible applications of such nanocomposites for other nanotechnological purposes, including biomedical applications.

### 3.2. Photothermal Bioimaging Sensors

A new approach to engineer multimodal core-shell NPs with a stably doped conductive polymer shell in biological environments was described. This method was achieved by making a densely packed polymer brush rather than changing its molecular structure. PANI was used as a model compound owing to its high-intensity near-infrared (NIR) absorption. It was grafted onto magnetic NPs via a polydopamine intermediate layer. Remarkably, at pH 7, its conductivity is ca. 2000× higher than that of conventional PANI nanoshells. Similarly, its NIR absorption is enhanced by two orders of magnitude, making it ideal for photothermal imaging and therapy. Another surprising finding is its non-fouling property, which even outperforms that of polyethylene glycol. This platform technology is also expected to open exciting opportunities in engineering stable conductive materials for electronics, imaging, and sensing.

Li et al. [42] developed a technology to synthesize conducting polymers with high resistance to dedoping and well-maintained electro-optical properties in physiological environments [21]. In contrast to prior attempts based on the chemical modification of monomers (a complex procedure with limited success), they achieved stable doping by engineering a dense brush polymer layer that is capable of trapping dopants with high affinity.

**Figure 5:**
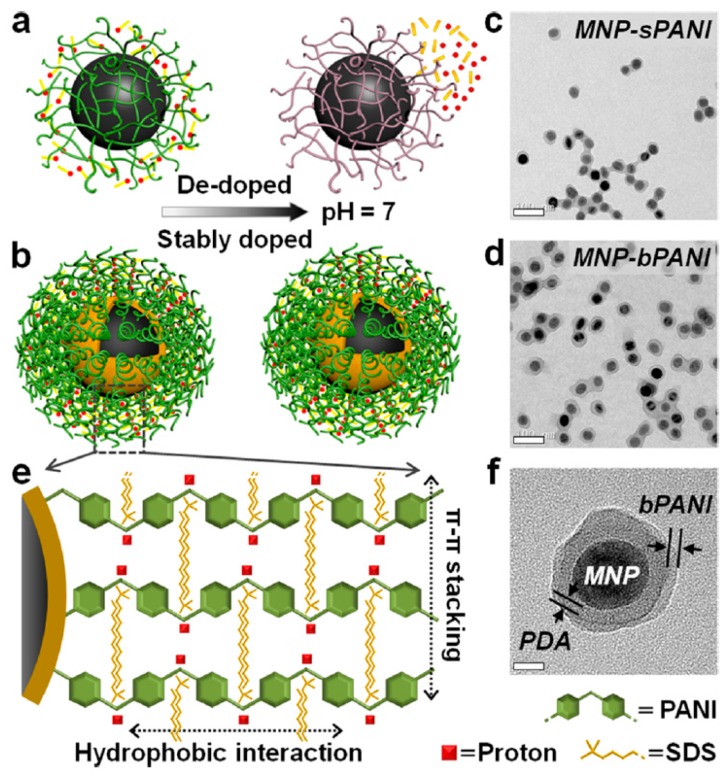
Design of stably doped PANI nanoshells. (**a**) Schematic illustrating conventional loosely packed PANI (spaghetti-like). The PANI layer loses its signature properties owing to dopant leaching at neutral pH; (**b**) Densely packed brush PANI with excellent doping stability; (**c**,**d**) Transmission electron microscope (TEM) showing the successful coating of PANI nanoshells on MNPs. Scale bar, 100 nm; (**e**) Diagram illustrating a high-density PANI brush polymerized on an intermediate PDA layer. Protons (red dots) and SDS (gold wires) are stably trapped owing to the strong interactions between the conjugate polymer chains and dopants; (**f**) TEM showing PDA-PANI dual shell layers on a MNP. Scale bar, 20 nm. Reprinted with permission from [42]. Copyright 2015, American Chemical Society. MNPs: magnetic NPs; PDA: polydopamine; SDS: sodium dodecyl sulfate.

As shown in Figure 5, stronger interactions between conjugated polymers and dopant ions can be expected if the polymer chains are densely packed because of the increased interactions between adjacent dopant ions and aniline segments.

To demonstrate the application of magneto-optical magnetic NPs (MNPs)-PANI in molecular imaging, the NPs were functionalized with a small-molecule ligand targeting prostate-specific membrane antigen (PSMA), one of the most specific biomarkers expressed in prostate tumor epithelial cells and an attractive target for imaging and therapeutic interventions [43]. bPANI’s NIR absorption is 2-fold higher than that of sPANI, thus representing an excellent NIR nanoprobe for photothermal imaging and therapy. A further surprising finding of the stably doped bPANI is its antifouling capability, even outperforming that of the gold-standard coating material, polyethylene glycol (PEG). Combining the electrical, optical, and interfacial properties of the stably doped conducting polymers, we envision that this simple and general approach will inspire a broad spectrum of applications in electronics, bioimaging, sensing, coating, and smart textiles.

### 3.3. Photoelectrochemical Biosensors

Recently, PANI has demonstrated its feasibility as an active component in many photoelectrochemical (PEC) devices (e.g., solar cells) owing to its unique electrical and optical properties, such as charge storage capacity, electron trapping capability, and photoelectric conversion performance [44]. For instance, the electrochromic performance of PANI can enhance light absorption in the visible region, hence producing more photogenerated electrons [45]. In addition, the conjugation structure of PANI molecules can capture more photoexcited electrons, retarding the recombination between photogenerated charges. Accordingly, modifying TiO_2_ with PANI is an effective strategy to improve the photocatalytic performance and shift the spectral absorption toward the visible region [46].

**Figure 6:**
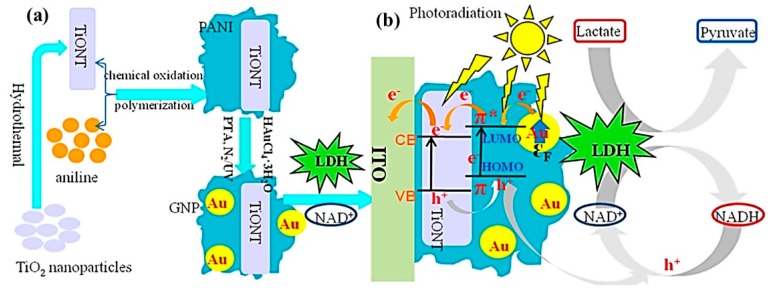
(**a**) Schematic illustration of the preparation of TiONT-PANI-GNPs ternary composites; (**b**) surface plasmon resonance (SPR)-enhanced photoelectrochemical (PEC) detection of lactate at the TiONT-PANI-GNPs|LDH|NAD+|ITO (indium tin oxide) coupling with PEC coenzyme regeneration. Reprinted with permission from [47]. Copyright 2015, American Chemical Society. LDH: lactate dehydrogenase.

A novel functional composite composed of TiO_2_ NTs (TiONTs), PANI, and GNPs was synthesized following the procedure illustrated in Figure 6a [47]. Aniline was initially polymerized on TiONTs via an oxidative polymerization method. 12-Phosphotungstic acid (PTA; PW_12_O_40_^3−^) was then modified on the PANI-TiONT surface, and the photochemically reduced PTA acted as a localized reducing agent for the deposition of GNPs on PANI-TiONT. A PEC biosensor for L-lactate was developed by immobilizing lactate dehydrogenase (LDH) and NAD+ on the composite-modified indium tin oxide (ITO) electrode. By integrating the surface plasmon resonance (SPR) effect of the GNPs and the excellent PEC performance of the PANI, the ternary composite transformed NADH back to NAD+ in a highly efficient manner to satisfy the LDH catalytic circle, improving the biosensor performance for the detection of lactate.

The PEC and electrochemical results demonstrated that the catalytic capability of LDH could be well maintained in the ternary composite. The TiONT-PANIGNPs-modified ITO electrode exhibited a stronger PEC response than the TiONTs- and TiONT-PANI-modified ITO because of the SPR-enhanced effect of GNPs and electrochromic performance of the PANI. TiONT-PANI-GNPs|LDH|NAD+-modified ITO as the proposed PEC biosensor showed excellent analytical performance for the detection of L-lactate, which was ascribed to the extraordinary PEC properties and biocompatibility of the ternary composite. In addition, the n-type TiONTs and p-type PANI can form a p–n heterojunction, which could increase the separation of the photoexcited charges, retard their recombination, and further increase the photocurrent of the PEC biosensor.

## 4. Poly(3,4-ethylenedioxythiophene) (PEDOT) Nanomaterials

PEDOT was developed as a new polythiophene (PT) derivative at the Bayer AG research laboratories in Germany during the late 1980s [48,49]. This polymer was initially improved to provide a soluble conducting polymer that lacked the existence of the α,β- and β,β-coupling within the polymer backbone. PEDOT, which was prepared by standard oxidative or electrochemical polymerization methods, was found to be an insoluble polymer but demonstrated some interesting properties, such as a high conductivity (ca. 300 S/cm). PEDOT was found to be transparent in thin film and exhibited excellent stability in the oxidized state [50]. The limitation of PEDOT such as solubility and stability was overcome by adding water-soluble polyelectrolyte, poly(styrene sulfonic acid) (PSS) which plays as the charge-balancing dopant during polymerization, resulting into the PEDOT/PSS. The mixture combined to PEDOT and the PSS electrolyte showed excellent physical properties for film-forming such as a water-solubility, high conductivity (ca. 10 S/cm), high visible light transmittance, and excellent physical stability [51]. The PEDOT/PSS thin film can be heated in air at 100 °C for over 1000 h with only a minimal change in conductivity.

Reports of the preparation of PEDOT NPs have been limited owing to the comparatively low solubility of the 3,4-ethylenedioxythiophene (EDOT) monomer in aqueous solution. There are several synthetic processes for preparing the PEDOT NPs by seed, dispersion, and emulsion polymerization methods.

(i) In 2004, Im et al. reported the fabrication of PEDOT NP using dodecylbenzenesulfonic acid (DBSA), a doping agent and surfactant, to form a micellar solution. Two different oxidants, such as FeCl_3_ and ammonium persulfate (APS), were utilized to polymerize the EDOT monomer in aqueous solution. The product was fairly spherical, and its diameter ranged from 35 to 100 nm. The highest conductivity of the PEDOT NP was evaluated to be 50 S/cm owing to the strong fascination of the oxidant-anionic surfactant in FeCl_3_-DBSA [52].
(ii) PEDOT core/shell and hollow nanoparticles with 100 nm polystyrene (PS) as the core material have been fabricated by dispersion polymerization process [53,54]. The PS was removed by DBSA as the surfactant to improve the stability of the PS [54]. It was supposed that hydrophobic alkyl chains, as oil phase, of the surfactant remained on the surface of the PS particles, whereas the sulfonic acid group was positioned toward the water phase. The EDOT monomer was absorbed onto the surface of the PS particle when the EDOT monomer was injected and instantly polymerized by the addition of the APS oxidant. The PS-PEDOT core-shell structure was distinctly characterized by TEM.
(iii) Seed polymerization is also an attractive methodology to prepare PEDOT NPs. Armed et al. reported the fabrication of PEDOT-coated PS latex using seed polymerization [55,56]. Polyvinylpyrrolidone (PVP)-stabilized PS latex was used as the core seed because PS particles provide narrow particle size distributions and the polymerization was proceeded by adding ferric tris(p-toluenesulfonate) at 85 °C in aqueous media. The PEDOT/PS core-shell materials were demonstrated by removing the core parts, PS.
(iv) Fabrication methods for one-dimensional (1-D) nanostructures of PEDOT, such as nanorods, NTs and NWs, have been suggested via various synthetic processes. 1-D PEDOT nanomaterials have been reported using microemulsion polymerizations, template free-methods, and electrochemical polymerizations. The fabrication of PEDOT nanofibers, NTs and NWs has mainly been used for the anodic aluminum oxide (AAO) membrane, so called hard-template method, because of AAO’s numerous advantages, such as its rigid shape, uniform diameter, and various pores sizes [57,58,59,60].

### 4.1. Glucose Biosensors

**Figure 7:**
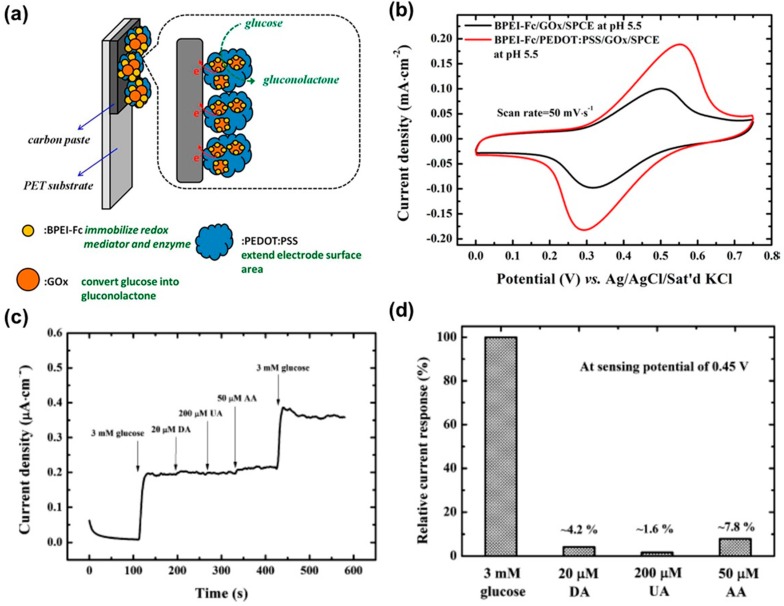
(**a**) Schematic diagram of the BPEI-Fc/PEDOT:PSS/GOx/SPCE; (**b**) CVs of the BPEI-Fc/GOx/SPCE and BPEI-Fc/PEDOT:PSS/GOx/SPCE in PBS solution, pH 5.5 at a scan rate of 0.02 V/s; (**c**) Amperometric i-t response of the BPEI-Fc/PEDOT:PSS/GOx/SPCE to the injection of 3 mM glucose and three common interfering species: 0.02 mM DA, 0.20 mM UA, and 0.05 mM AA. The working potential was 0.45 V; (**d**) Current response of glucose (set as 100%), DA, UA, and AA of the BPEI-Fc/PEDOT:PSS/GOx/SPCE. Reprinted with permission from [65]. Copyright 2013, American Chemical Society. BPEI-Fc: polyethylenimine binding with ferrocene; SPCE: screen-printed carbon electrode; GOx: glucose oxidase.

Glucose sensors are critical monitoring tools for diabetes. Accordingly, accurate and rapid glucose determination is significant in the field of clinical and food analysis. Whereas blood glucose levels in healthy humans range from 4 to 8 mM, diabetics have a considerably larger range of approximately 2 to 30 mM. This large variation results in serious health problems. The glucose levels in the human body divides into two different phenomena: (i) low glucose levels (hypoglycemia) lead to fainting, coma, and even death; (ii) whereas high glucose levels (hyperglycemia) have a bad influence on the the eyes, kidneys, nerves, and blood vessels [61]. Glucose sensors using PEDOT CP transistor have been commonly fabricated by the immobilization of glucose oxidase enzyme (GOx) on the surface of the PEDOT transistor. In 2004, Zhu et al. [62] demonstrated a glucose biosensor with an organic electrochemical transistor (OECT), in which the commercially available CP PEDOT:PSS was the semiconducting layer of the transistors, capable of sensing glucose in a neutral pH buffer solution. In 2007, this work was expanded when a variety of concentrations of glucose solutions were blended with GOx and detected with micromolar sensitivity. The sensor was demonstrated by a CP transistor with a channel composed of PEDOT:PSS and a Pt gate electrode. This sensor is able to detect glucose well within the clinical range of glucose levels in human saliva [63]. In 2013, Liao et al. [64] reported that the sensitivity of glucose sensors based on OECTs is enhanced by GOx embedded in graphene or reduced graphene oxide (rGO)-modified gate electrodes. The optimized device provides linear responses to glucose in the range of 10 nM to 1 μM and with a MDL as low as 10 nM. The selectivity of the device was systematically studied for the first time. The device selectivity is dramatically improved when the gate electrode is modified with biocompatible polymers (Nafion or chitosan). The interference by uric acid and ascorbic acid is almost negligible for practical applications. Wang et al. [65] prepared redox polymer nanobeads branched by polyethylenimine binding with ferrocene (BPEI-Fc) using a simple chemical reaction process (Figure 7). The nanobeads were hydrophilic redox and were mixed with glucose oxidase (GOx) for drop-coating onto a screen-printed carbon electrode (SPCE). Cyclic voltammetry analysis was introduced to characterize the electrochemical properties of the BPEI-Fc/GOx/SPCE prepared under different conditions. Based on the CV data, the synthetic process of the BPEI-Fc/GOx/SPCE was optimized. The performance of a redox polymer nanobead with enzyme electrode (BPEI-Fc/PEDOT:PSS/GOx/SPCE) was improved by incorporating conductive PEDOT:PSS/GOx/SPCE which exhibits high glucose oxidation currents. The glucose sensing sensitivity of the BPEI-Fc/PEDOT:PSS/GOx/SPCE was calculated up to 66 μA·mM^−1^·cm^−2^, which is 2.5 times higher than that of a device without PEDOT:PSS. This indicates that BPEI-Fc/PEDOT:PSS/GOx/SPCE provides a more efficient oxidizing pathway for glucose. The interference property was demonstrated by a mixture consisting of glucose and three common interfering species (ascorbic acid, dopamine, and uric acid) at physiological levels. The interferences of dopamine (4.2%) and ascorbic acid (7.8%) from the electrodes were acceptable, while the current response to uric acid (1.6%) was negligible compared to the current response to glucose. More recently, Yang et al. [66] reported a novel method for the fabrication of GOx entrapped in CP nanofibers on the surface of a Pt microelectrode. GOx entrapped in PEDOT was prepared in films (PEDOT F-GOx) and in nanofibers (PEDOT NFs-GOx). The electrical properties, sensitivity, and longevity of each type of biosensors were measured at the same conditions (polarization potentials of +300 and +700 mV vs. Ag/AgCl). The significant reduction in impedance for PEDOT NFs-GOx and F-GOx was calculated to compare the sensitivity of the PEDOT NFs-GOx biosensors. At both polarization potentials, the PEDOT NFs-GOx biosensors showed a larger amperometric response to the glucose samples than that of the PEDOT F-GOx biosensors.

### 4.2. Dopamine Biosensors

**Figure 8:**
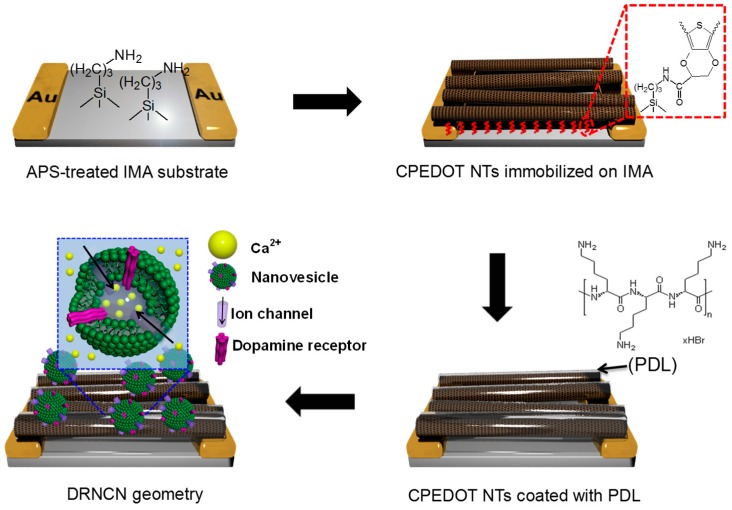
Schematic illustrations of the steps for constructing the DRNCN geometry. Reprinted with permission from [70]. Copyright 2014, Nature Publishing Group. APS: ammonium persulfate; IMA: interdigitated microelectrode array; PDL: poly-d-lysine.

Dopamine is a significant neurotransmitter found in blood samples at concentrations between 0.01 and 0.1 μM that mediates the transmission of messages in the central nervous system. Its deficiency may cause various neurological disorders; Parkinson’s disease, Alzheimer’s disease, and schizophrenia which are always associated with the abnormal metabolism of dopamine. Vasantha et al. [67] used a PEDOT-modified glassy carbon electrode and measured various combinations of dopamine and ascorbate anions. Well-separated voltammetric peaks were observed for dopamine and ascorbate anions with a peak separation of 0.170 V. Tang et al. [68] examined a dopamine sensor utilizing OECTs based on PEDOT:PSS with different gate electrodes, including graphite, Au, and Pt electrodes. The sensitivity of the OECTs to dopamine depends on the gate electrode and operation voltage. A Pt gate electrode was characterized at a gate voltage of 0.6 V and exhibited high sensitivity. The MDL of the device to dopamine is approximately 5 nM. Further research in the same group investigated OECTs used as highly sensitive and selective dopamine sensors. The selectivity of the OECT-based dopamine sensors was significantly improved by applying a coating of the biocompatible polymer Nafion or chitosan on the graphene/Pt or rGO/Pt gate electrodes. Furthermore, interference molecules, such as uric acid and ascorbic acid, are effectively eliminated after the modification with Nafion. The MDL of the devices to dopamine is as low as 5 nM [69]. Recently, Park et al. [70] proposed the fabrication process for a high-performance dopamine biosensor based on the FET system incorporated with a dopamine receptor-containing nanovesicle-immobilized carboxylated PEDOT NTs (DRNCNs) (Figure 8). Nanovesicles containing a human dopamine receptor D1 (hDRD1) were successfully prepared from HEK-293 cells, stably expressing hDRD1. The hDRD1-involving nanovesicles were used for the gate-potential modulator on the PEDOT NT transistors, providing high-performance responses toward the dopamine molecule owing to their uniform and monodisperse morphologies. The DRNCNs were integrated into a liquid-ion gated FET system via immobilization processes, leading to high sensitivity and excellent selectivity toward dopamine in a liquid state. The MDL of FET DRNCNs was as low as 10 pM in real-time responses which was a 10 times more sensitive result compared to conventional dopamine biosensors. More interestingly, the response time of the FET-type DRNCN biosensor toward dopamine was very rapid (>1 s) and the biosensor had an excellent selectivity in serum.

## 5. Conclusions

This review introduced various nanobiosensors, including human sense mimicking sensors, aptasensors, immunoassays, H_2_O_2_ biosensors, glucose biosensors, and FET biosensors, based on CP nanomaterials, such as PPy, PANI, and PEDOT, owing to their large surface areas and inherent conductivity. We also proposed recently developed fabrication processes for CP nanomaterials with different shapes and sizes, which resulted in attractive transducers or electrodes. Responses from those biosensors were rapid and sensitive toward target molecules. In particular, CP nanomaterials were integrated into the liquid-ion gated FET system, leading to real-time responses with high sensitivity and selectivity. However, although those CP nanomaterials have been used for biosensors, the sensing performance can still be improved through novel methods for surface modification and three-dimensional structures with enhanced surface areas.

## Abbreviations

The following abbreviations are used in this manuscript:

CP	Conducting polymer
FET	Field-effect transistor
PPy	Polypyrrole
PANI	Polyaniline
PEDOT	Poly(3,4-ethylenedioxythiophene)
CNT	Carbone nanotube
MDL	Minimum detectable level
CPNT	Carboxylated polypyrrole nanotube
B-nose	Bioelectronic nose
hORs	Human olfactory receptor
NBE	Nanobioelectronic tongue
PTC	Phenylthiocarbamide
VEGF	Antivascular endothelial growth factor
NDFLG	Nitrogen-doped few-layer graphene
NW	Nanowire
p*I*	Isoelectric point
NC	Nanocomposite
MBA	Myoglobin-binding aptamer
Mb	Myoglobin
OPPy	Over-oxidized PPy
SPE	Screen printed electrodes
GO	Graphene oxide
rGO	Reduced graphene oxide
MEMS	Microelectromechanical system
PDMS	Poly(dimethylsiloxane)
CV	Cyclic voltammetry
GNPs	Gold nanoparticles
GOx	Glucose oxidase
AAO	Anodic aluminum oxide
hDRD1	Human dopamine receptor D1
